# COVID-19 information retrieval with deep-learning based semantic search, question answering, and abstractive summarization

**DOI:** 10.1038/s41746-021-00437-0

**Published:** 2021-04-12

**Authors:** Andre Esteva, Anuprit Kale, Romain Paulus, Kazuma Hashimoto, Wenpeng Yin, Dragomir Radev, Richard Socher

**Affiliations:** 1 Salesforce Research, Palo Alto, CA USA; 2 Yale University, New Haven, CT USA

**Keywords:** Medical research, Health care, Publishing

## Abstract

The COVID-19 global pandemic has resulted in international efforts to understand, track, and mitigate the disease, yielding a significant corpus of COVID-19 and SARS-CoV-2-related publications across scientific disciplines. Throughout 2020, over 400,000 coronavirus-related publications have been collected through the COVID-19 Open Research Dataset. Here, we present CO-Search, a semantic, multi-stage, search engine designed to handle complex queries over the COVID-19 literature, potentially aiding overburdened health workers in finding scientific answers and avoiding misinformation during a time of crisis. CO-Search is built from two sequential parts: a hybrid semantic-keyword retriever, which takes an input query and returns a sorted list of the 1000 most relevant documents, and a re-ranker, which further orders them by relevance. The retriever is composed of a deep learning model (Siamese-BERT) that encodes query-level meaning, along with two keyword-based models (BM25, TF-IDF) that emphasize the most important words of a query. The re-ranker assigns a relevance score to each document, computed from the outputs of (1) a question–answering module which gauges how much each document answers the query, and (2) an abstractive summarization module which determines how well a query matches a generated summary of the document. To account for the relatively limited dataset, we develop a text augmentation technique which splits the documents into pairs of paragraphs and the citations contained in them, creating millions of (citation title, paragraph) tuples for training the retriever. We evaluate our system (http://einstein.ai/covid) on the data of the TREC-COVID information retrieval challenge, obtaining strong performance across multiple key information retrieval metrics.

## Introduction

The evolution of the SARS-CoV-2 virus, with its unique balance of virulence and contagiousness, has resulted in the COVID-19 pandemic. Since December 2019, the disease threatens exponential spread across our society, catalyzed by a modern air and road transportation system, along with dense urban centers where close contact amongst people yielded hubs of viral spread.

Global efforts have arisen in an attempt to quell the spread of the virus. National governments have shut down entire economic sectors, enforcing stay-at-home orders for many people. Hospitals have restructured themselves to cope with an unprecedented influx of intensive care unit patients, sometimes growing organically to increase their number of beds^1^. Institutions have adjusted their practices to support efforts—repurposing assembly lines to build mechanical ventilators^2^, delaying delivery of non-COVID-related shipments^3^, creating contact-tracing mobile apps^4^ and "digital swabs”^5^ to track symptoms and potential spread. Pharmaceutical enterprises and academic institutions have invested significantly in developing vaccines and therapeutics^6^, while deeply studying both COVID-19 and SARS-CoV-2.

The health impacts of this crisis have been matched only by the economic backlash to society. Hundreds of thousands of small businesses have shut down, entire industrial sectors have been negatively impacted^7^, and tens of millions of workers have been laid off or furloughed^8^. Even after our global society succeeds at controlling the virus’s spread, we will be faced with many challenges, including re-opening our societies, lifting stay-at-home orders, deploying better testing, developing vaccines and therapeutics, aiding the unemployed and out-of-business, etc.

The global response to COVID-19 has yielded a growing corpus of scientific publications—increasing at a rate of thousands per week—about COVID-19, SARS-CoV-2, other coronaviruses, and related topics^9^. The individuals on the front lines of the fight—healthcare practitioners, policy makers, medical researchers, etc.—will require specialized tools to keep up with the literature.

CO-Search is a cascaded retriever-ranker semantic search engine that takes complex search queries (e.g. natural language questions), and retrieves scientific articles strictly over the coronavirus-related literature. CO-Search indexes content from over 400,000 scientific papers made available through the COVID-19 Open Research Dataset Challenge (CORD-19)^9^—an initiative put forth by the US White House and other prominent institutions in early 2020. The goal of this line of work is to offer an alternative, scientific search engine, designed to limit misinformation in a time of crisis.

We evaluate CO-Search on data from the TREC-COVID challenge^10^—a five-round information retrieval (IR) competition for COVID-19 search engines—using several standard IR metrics: normalized discounted cumulative gain (nDCG), precision with N documents (P@N), mean average precision (MAP), and binary preference (Bpref). For full details see the “Methods” section. TREC-COVID considers IR system submissions that are either *manual*—in which queries and retrieved documents may be manually adjusted by a human operator—or *automatic* (such as CO-Search)—in which they may not. A third category is accepted in Rounds 2–5, of type *feedback*, in which systems are trained with supervision from the annotations of prior rounds. Submissions compete on a predefined set of topics, and are judged using a number of metrics, including those listed above. Expert human annotators provide relevance judgments on a small set of topic–document pairs, which are included, together with non-annotated pairs, in the evaluation.

The CORD-19^9^ coronavirus-related literature corpus, primarily from PubMed, mostly published in 2020, has quickly generated a number of data science and computing works^11^. These cover topics from IR to natural language processing (NLP), including applications in question answering^12^, text summarization, and document search^10^.

In 2020, more than 20 organizations have launched publicly accessible search engines using the CORD-19 corpus. For instance, Neural Covidex^13^ was constructed from various open source information-retrieval building blocks, as well as a deep learning transformer^14^ finetuned on a machine-reading comprehension dataset (MS MARCO)^15^ to predict query-document relevance, for ranking. SLEDGE^16^ extends this by using SciBERT^17^—the scientific text-trained version of the prominent BERT^18^ NLP model—also finetuned on MS MARCO, to re-rank articles retrieved with BM25.

One of the first question–answering systems built on top of the CORD-19 corpus is CovidQA (http://covidqa.ai), which includes a small number of questions from the CORD-19 tasks^12^. CAiRE is a multi-document summarization system^19^ which works by first pre-training on both a general text corpus^20,21^ and a biomedical review dataset, then finetuning on the CORD-19 dataset.

One of the applications of the corpus has been Named Entity Recognition (NER). Wang et al.^22^ introduce the COVID-NER corpus, which includes 75 fine-grained entity types, both conventional (e.g., genes, diseases, and chemicals) and corpus-specific (e.g., viral proteins, coronaviruses, substrates, and immune responses). Ahamed and Samad perform a network analysis of the corpus^23^, in which they use word associations to identify the phrases that co-occur with the most medically relevant keywords. This allows them to identify information about different antiviral drugs, pathogens, and pathogen hosts, as well as proteins and medical therapies, as to how they are connected to the central topic of “coronavirus”.

Broader surveys^11^ of the COVID-19-related literature have already arisen, covering a wider range of research perspectives including molecular, clinical, and societal factors. Roberts et al. (2020)^10^ offers an in-depth analysis of the TREC-COVID competition structure, including the notable differences in IR systems for pandemics, which deviate substantially from typical IR systems. They address key questions around COVID-19-specific IR systems, including: How are topics different from typical web-based search? What is the appropriate search content? How to deploy quickly? What are the appropriate IR modalities? How to customize IR systems for pandemics? Can existing data be leveraged? How to best respond to the rapidly growing literature corpus? How to evaluate systems? And so forth. COVID search engines differ from more general neural IR engines^24,25^ because of the relatively limited and focused, and also rapidly changing collection of documents. Another recent system paper from the challenge is ref. ^26^, in which the authors describe an ensemble system that combines more than 100 IR methods, including lexical rankers, embeddings, as well as relevance feedback. Our proposed method builds on these insights by selectively choosing three deep-learning methods and showing how they each enhance COVID-specific scientific search.

## Results

### Dataset

To quantitatively evaluate the effectiveness of our search engine, we combine the CORD-19 corpus with the TREC-COVID competition’s evaluation dataset. The evaluation dataset consists of topics, along with relevance judgments which assign topic–document pairs into one of the following groups: irrelevant, partially relevant, or relevant. See Table 1 for example topics. The relevance judgments are determined by human experts in related fields (biology, medicine, etc.).

**Table 1:** Sample TREC COVID topic Search topics are tuples consisting of a query, a question, and a narrative, each sequentially more detailed.

Query	coronavirus drug repurposing
Question	which SARS-CoV-2 proteins–human proteins interactions indicate potential for drug targets. Are there approved drugs that can be re-purposed based on this information?
Narrative	Seeking information about protein–protein interactions for any of the SARS-CoV-2 structural proteins that represent a promising therapeutic target, and the drug molecules that may inhibit the virus and the host cell receptors at entry step.
Example articles	• Re-purposing approved drugs as inhibitors of SARS-CoV-2 S-protein from molecular modeling and virtual screening.
	• Drug repurposing using computational methods to identify therapeutic options for COVID-19.
	• Virtual screening, ADME/Tox predictions and the drug repurposing concept for future use of old drugs against the COVID-19.
Query	coronavirus mental health impact
Question	How has the COVID-19 pandemic impacted mental health?
Narrative	Includes increasing/decreasing rates of depression, anxiety, panic disorder, and other psychiatric and mental health conditions.
Example articles	• Early impacts of the COVID-19 pandemic on mental health care and on people with mental health conditions.
	• Impact on mental health care and on mental health service users of the COVID-19 pandemic.
	• Mental Health and the COVID19 Pandemic.

Here we show two such topics, along with example articles which have been judged by experts as being relevant to the given topics.

The U.S. White House, along with the U.S. National Institutes of Health, the Allen Institute for AI, the Chan-Zuckerberg Initiative, Microsoft Research, and Georgetown University recently prepared the CORD-19 Challenge in response to the global crisis. As of February 2021, this resource consists of over 400,000 scientific publications (up from 29,000 at the challenge inception in February 2020) about COVID-19, SARS-CoV-2, and earlier coronaviruses^9^.

This challenge represents a call to action to the artificial intelligence (AI) and IR communities to "develop text and data mining tools that can help the medical community develop answers to high priority scientific questions”. It is currently the most extensive coronavirus literature corpus publicly available.

To build on CORD-19, the Text Retrieval Conference (TREC) recently partnered with the National Institute of Standards and Technology (NIST), to define a structured and quantitative evaluation system for coronavirus IR systems. The TREC-COVID challenge^10^ is composed of five successive rounds of evaluation on 30–50 topics. The first round includes 30 topics. Each subsequent round takes the prior round’s topics and adds five new ones.

Each topic is represented as a tuple consisting of a query, a question, and a narrative, with an increasing amount of detail in each. IR systems must retrieve up to 1000 ranked documents per topic from the CORD-19 publications, and are evaluated on many metrics. See the “Methods” section for further details.

### System architecture

CO-Search consists of a retriever, which returns a sorted subset of documents from the general corpus, a re-ranker, which further sorts them, and an offline pre-processing step known as document indexing, which parses documents via a combination of deep learning and keyword-based techniques to make them both semantically and syntactically searchable at scale. This process converts pieces of raw text into high-dimensional vector representations, such that one vector’s proximity to another indicates similar content. The full system is shown in Fig. 2.

**Fig. 1:**
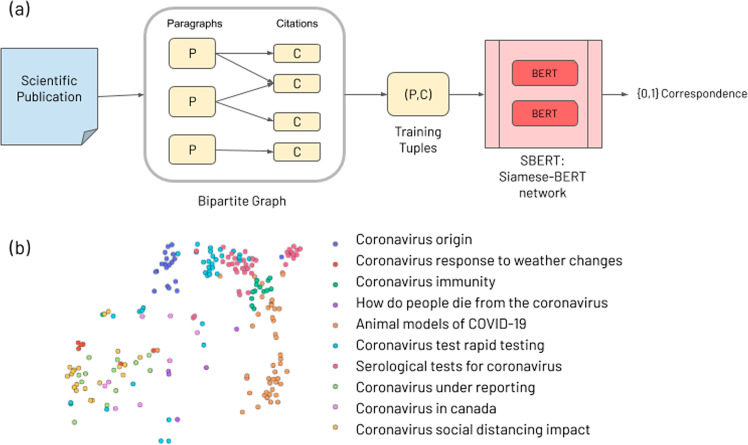
Semantic search: learning to match queries to publication titles. **a** Documents are split into paragraphs and the citations included in them to form a bipartite graph that induces training tuples (*p*, *c*). These are fed to a Siamese-BERT (SBERT) model trained to discern if a citation is contained in a given paragraph. This process makes SBERT match user search queries to scientific publication titles. **b** t-SNE visualization of the SBERT embeddings of entire documents, each denoted by a single point. Their color represents the topic to which they are most closely matched. Notably, queries pertaining to the same topic tend to cluster together.

**Fig. 2:**
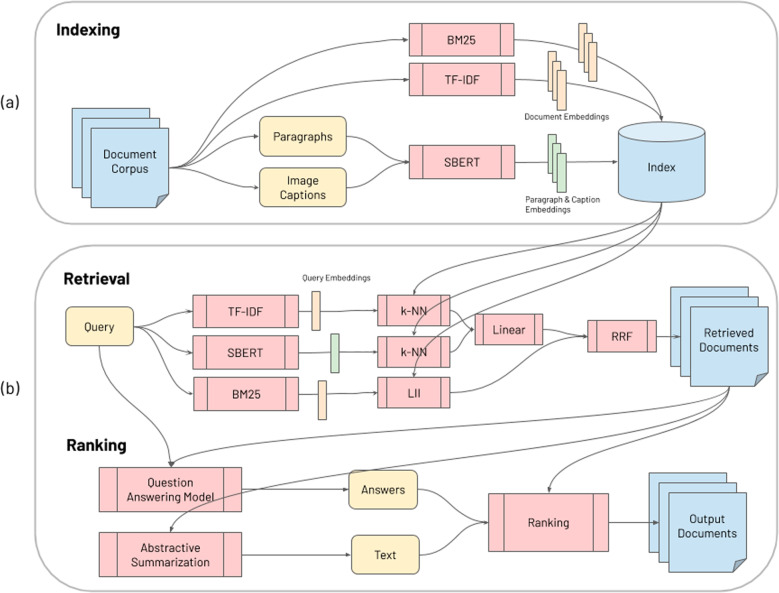
Cascaded semantic search engine architecture. **a** Indexing: Raw documents are processed into a searchable format. Documents are split into paragraphs and image captions, embedded with an SBERT deep learning model, and stored into an index. The raw documents are also embedded with two-keyword-based models (TF-IDF and BM25). **b** Retrieval and re-ranking: The system computes a linear combination of TF-IDF and SBERT retrieval scores, then combines them with the retrieval scores of BM25 using reciprocal rank fusion^31^, to generate a sorted candidate list. k-Nearest-Neighbors are used for TF-IDF and SBERT, and the Lucene Inverted Index is used for BM25. The retrieved documents and the query are parsed using a question answering model and an abstractive summarizer prior to being re-ranked based on answer match, summarization match, and retrieval scores.

The index is created by processing documents in three ways: a deep learning model called Siamese-BERT (SBERT^27^) embeds single paragraphs and image captions, and two keyword-based models (TF-IDF, BM25^28^) vectorize entire documents (see Fig. 2a). SBERT is an extension of the widely used BERT^18^ language representation model which uses two BERT models with tied network parameters. It has been shown to be superior to BERT in semantic similarity search^27^ by being significantly more computationally efficient at learning correspondences between sentences. For instance, finding the most similar pair of sentences, using BERT, in a collection of *n* = 10,000 sentences would require each possible pair to be fed into the network, one sentence at a time, yielding *n*(*n* − 1)/2 = 49,995,000 inference computations, or about 65 h on an NVIDIA V100 GPU. In contrast, SBERT reduces this to 10,000 inference computations and the computation of cosine similarity distances between them, yielding about 5 s of compute time. SBERT is trained to take a short text string and a longer text document and output the correspondence between the two (i.e. their similarity) as a real-valued number between 0 and 1. In this use case, semantic embeddings from the SBERT model face the challenge of working with a relatively small number of long documents. We account for this by pre-training SBERT on a large, synthetic dataset of millions of training examples, constructed as follows. We split documents into paragraphs, extract the titles of the citations of each paragraph, and form a bipartite graph of paragraphs and citations with edges implying that a citation *c* came from a paragraph *p*. We use the graph to form tuples ((*p*, *c*) s.t.*c* ∈ *p*) for training SBERT to predict if a title was cited by a paragraph. Additionally, we generate an equivalent number of negative training samples of incorrect tuples ((*p*, *c*) s.t.*c* ∉ *p*). The full pipeline for this step is shown in Fig. 1a.

The structure of the embedded space is such that proximal queries and documents share semantic meaning. Visualizing this reveals a human-understandable clustering of documents and topics. Figure 1b shows a two-dimensional t-SNE^29^ plot—an effective method for visualizing high-dimensional data—of the embedded space, with different colors representing topics of TREC-COVID, and points representing documents. We can observe that semantically similar documents cluster by topic.

Document retrieval (Fig. 2b, top row)—which returns a list of the top 1000 documents for a query—is accomplished by fusing the returned lists of the SBERT, TF-IDF, and BM25 models. SBERT allows for variable-length queries and documents to be embedded into the same vector space (the multi-dimensional internal representation of the data, by the model), in order to model semantic proximity and enable k-nearest-neighbor (kNN) retrieval. We use approximate kNN retrieval using the Annoy framework (https://github.com/spotify/annoy), to account for the large number of paragraphs parsed by SBERT. TF-IDF and BM25 independently return two document lists (TF-IDF uses kNN with cosine distance; BM25 uses a Lucene inverted index^30^, built with Anserini) that either share in the most unique keywords of the query (TF-IDF) or share many of the same keywords as the query (BM25-Anserini)^28^.

These three lists are then combined by first linearly fusing the SBERT list with the TF-IDF list, then using reciprocal rank fusion (RRF)^31^ to merge this with the BM25 list. This retrieval process returns the top 1000 documents as a function of their semantic and syntactic distance to the query.

Document re-ranking (Fig. 2b, bottom row) takes this set of documents, runs them through both a question–answering module (QA) and a summarizer, then ranks the documents by a weighted combination of their original retrieval scores, the QA output, and the summarizer output. Whereas standard question answering systems generate answers, our model extracts multiple answer candidates (text spans) from the paragraphs of the retrieved documents. This is accomplished by taking the query and the retrieved paragraphs, and using a sequential paragraph selector^32^, to filter for a set of paragraphs that, when combined, could answer the query. Specifically, the model uses multi-hop reasoning to model relationships between paragraphs, and selects sequentially ordered sets of them. It is pre-trained using a Wikipedia-derived dataset of 113k question–answer pairs and sentence-level supporting facts^33^, and further finetuned on a QA dataset built from PubMed^34^, for biomedical specificity. Once filtered, these sequential paragraph sets are fed into a reading comprehension model (trained on a standard question–answering dataset with topic structure similar to CORD-19^35^) to extract answer candidates.

In a parallel fashion, the summarizer generates a single abstractive summary from the retrieved documents. It is built in an encoder–decoder fashion, in which an encoder (BERT^18^) first embeds an entire document, and a decoder (a modified GPT-2 model^36^) converts this embedding into raw text, outputting a summary. To increase the probability that a generated summary matches (and thus, helps re-rank) the contents of the retrieved paragraphs, we tuned the model to generate short summaries of fewer than 65 words^37^.

Finally, the system uses the generated answers and summary to compute two scores for each retrieved document. The first measures the relevance of a document, given the query, and the second measures the degree to which any single document summarizes the entire set of retrieved documents. These two scores are combined with the original relevance scores to output a final ranked list of documents.

### Evaluation

We evaluate our system quantitatively using the CORD-19 document dataset and the topics and relevance judgments provided by TREC-COVID. The dataset contains five sets of topics, where each topic is represented as a (query, question, narrative) tuple. Relevance judgments—provided on a very small subset of all possible topic–document pairs—scores topic–documents as irrelevant, partially relevant, or relevant. These judgments have been iteratively gathered throughout the course of the five-round TREC-COVID competition, in which search engines submitted up to 1000 ranked documents per query, and the organizers pooled from amongst the most common topic–document pairs for judging (i.e. depth-*N* pooling, in which the top *N* documents from each response provided by the set of contributing systems are judged for relevance by human assessors^38^, with *N* ranging from 7 to 20 for the various rounds). The pool depths results in many relevant documents being missed. Though this labeling procedure is inherently sparse and somewhat biased, this is the best available method for evaluating IR systems, as obtaining relevance judgments on all possible topic–document pairs is infeasible.

In order to better evaluate our approach, we use a variety of IR metrics. Key amongst them are high-precision metrics such as nDCG, top-*N* precision, and MAP. The critical limitation with these is that their effectiveness relies on complete relevance judgments across all topic–document pairs. To account for this, an additional metric, Bpref, which is robust to missing relevance judgments, is considered. For full details, see the “Methods” section.

Our results on this data are shown in Table 2. We compare the performance of our system in two contexts. The first context is within the general set of submissions. This includes metric evaluations on all documents—annotated and non-annotated—and this includes ranking against the three possible system types in the competition: *manual*, *automatic*, and *feedback* systems. *Manual* submissions use human operators that can iteratively adjust the query or the retrieved documents to improve ranking. *Feedback* systems are trained using the relevance judgments of prior rounds. *Automatic* search engines may not do either. Strictly speaking, *feedback* systems are also automated (in that they do not use a human in the loop), though they have an inherent advantage over *automatic* systems and are thus considered separately. In the second context, we evaluate our system (and all others) strictly on relevance judgments, and we compare our automatic system strictly against other automatic systems. Specifically, we re-score every automatic system’s runs after removing non-judged topic–document pairs. To determine team rankings, we account for both multiple submissions per team, and for multiple submissions with the same score, assigning to each the highest one (i.e., if the top two scoring submissions for a metric have the same score, each would be ranked #1).

**Table 2:** TREC-COVID results.

	Score	Team rank	Score	Team rank
**Round 1**	**All submissions (144)**		**Automatic submissions (102)**	
	**All pairs (1.53M)**		**Judged pairs (8691)**	
Bpref	0.5176	2	0.5176	1
MAP	0.2401	13	0.4870	1
P@5	0.6333	19	0.8267	1
P@10	0.5567	21	0.7933	1
nDCG@10	0.5445	13	0.7233	1
**Round 2**	**All submissions (136)**		**Automatic submissions (73)**	
	**All pairs (2.20M)**		**Judged pairs (12,037)**	
Bpref	0.5402	2	0.5232	1
MAP	0.3487	1	0.5138	1
P@5	0.8000	3	0.8171	1
P@10	0.7200	3	0.7629	1
nDCG@10	0.6996	1	0.7247	1
**Round 3**	**All submissions (79)**		**Automatic submissions (32)**	
	**All pairs (5.14M)**		**Judged pairs (12,713)**	
Bpref	0.5665	7	0.5665	1
MAP	0.3182	7	0.5385	1
P@5	0.7800	14	0.8200	2
P@10	0.7600	12	0.7850	2
nDCG@10	0.6867	12	0.7065	2
**Round 4**	**All submissions (72)**		**Automatic submissions (28)**	
	**All pairs (7.10M)**		**Judged pairs (13,262)**	
Bpref	0.5887	7	0.5887	3
MAP	0.3436	10	0.5653	3
P@5	0.8222	14	0.8222	5
P@10	0.7978	12	0.8133	4
nDCG@10	0.7391	12	0.7449	6
**Round 5**	**All submissions (126)**		**Automatic submissions (49)**	
	**All pairs (9.56M)**		**Judged pairs (23,151)**	
Bpref	0.5253	13	0.5253	3
MAP	0.3089	14	0.4884	3
P@5	0.8760	13	0.876	3
P@10	0.8260	15	0.842	3
nDCG@10	0.7488	16	0.7567	4

Performance evaluation of the COVID-19 search engine on the five rounds of the TREC-COVID challenge dataset. Two contexts are considered. Context 1 (columns “All submissions, All pairs”) considers our search engine performance against all search engines—manual, feedback, and automatic engines—using both annotated and non-annotated topic-document pairs. Context 2 (“Automatic submissions, Judged pairs”) considers our search engine performance strictly against those in its class—automatic search engines, using topic–document pairs annotated by experts.

Each round builds on the previous rounds, adding five new topics, many documents, as well as new relevance judgments. As a result, Round 5 is the most complete round. In the first context (columns “All submissions, All pairs”), our system ranks in the top 21 (Table 2) across all rounds. In considering the rankings from Round 1 through Round 5, there is a pronounced improvement in rankings from Round 1 to Round 2, with a drop then plateau in performance from Rounds 3 to 5. The improvement from Round 1 to 2 can be explained by the judgment fraction—the percentage of relevance judgments goes up, increasing the performance across these metrics. This happens because metrics such as precision penalize search engines for retrieving relevant but non-annotated documents for a topic. Rounds 3–5 have sufficient relevance judgments from prior rounds to improve feedback systems, leading to a drop in the ranking.

In the second context, our system ranks in the top 6 across all metrics and all rounds, in the top 4 across all but four, and as the top 1 system across half of them. The stability in performance is largely due to the consistent judgment fraction (100%, implicitly), and the absence of *feedback* and *manual* systems, both of which improve with relevance judgments. This stability—evident also in the metrics—implies a system that is robust to increasing corpus size.

Of note, the availability of relevance judgments is quite sparse throughout all rounds, with Round 1 exhibiting a coverage of 0.57%, and Round 5 a coverage of 0.24%. This is precisely what motivates the use of the Bpref metric, which is robust to missing annotations, as evidenced by its consistency across contexts.

## Discussion

Here we present CO-Search, a scientific search engine over the growing corpus of COVID-19 literature. We train the system using the scientific papers of the COVID-19 Open Research Dataset challenge, and evaluate its performance using the data of the TREC-COVID competition on a number of key metrics, achieving strong performance across metrics and competition rounds. The system uses a combination of semantic and keyword-based models to retrieve and score documents. It then re-ranks these documents by using a Wikipedia-trained & PubMed-trained question–answering system, together with an abstractive summarizer, to modulate retrieval scores.

We perform an ablation study of our system using Round 5 data (first context) in order to examine the performance effects of its components (Table 3). This is done in two steps, first for the retriever, then for the re-ranker. For each, we analyze the metric performance of various components individually, and united. The retriever’s components (TF-IDF, BM25, SBERT) each perform poorly, but benefit from substantial synergy when united into the full retrieval pipeline (top half of Table 3). This occurs because keyword-based techniques, on their own, do not perform as well on queries in natural language. Similarly, semantic techniques tend to underweight the most salient keywords of a natural language query. Combined, these two techniques work well for this unique dataset. The retrieval subsystem accounts for most of the performance of the overall system. The addition of the re-ranker, with its two other deep learning modules (Q&A, summarizer) serve to further boost this performance on the order of 1–2% across the various metrics employed.

**Table 3:** Ablation study.

System	Bpref	MAP	P@5	P@10	nDCG@10
*Retrieval*					
SBERT	0.3594	0.1128	0.4640	0.4180	0.3658
TF-IDF	0.2567	0.0781	0.3320	0.3380	0.2567
BM25	0.4581	0.1313	0.2360	0.2300	0.2221
Retrieval (all above)	**0.5146**	**0.2987**	**0.8680**	**0.8200**	**0.7254**
*Re-Ranking*					
Retrieval + QA	0.5205	0.3075	0.8720	0.8210	0.7298
Retrieval + AS	0.5246	0.3049	0.8680	0.8235	0.7312
Retrieval + QA + AS	**0.5253**	**0.3089**	**0.8760**	**0.8260**	**0.7488**

We iteratively eliminate various pieces of the search engine in order to compute their effect on the system’s performance. In the retrieval subsystem (top half), Siamese-BERT semantic retrieval (SBERT) and keyword-based retrieval (TF-IDF, BM25) each perform substantially worse than the unified whole (Retrieval). In the re-ranker subsystem (bottom half), both the Question–Answering (QA) and Abstractive Summarization (AS) modules marginally boost the performance of the retrieval metrics.

Bold values indicate the top-scoring system for the given column’s metric.

We compare our system against three of the top-performing systems of Round 5, as shown in Table 4. As can be seen, no single system outperforms the rest across all metrics, indicating the possibility of forming hybrid systems that benefit from the strengths of each. The system *covidex*^13^ uses a transformer fine-tuned on the MedMARCO machine-reading comprehension dataset^16^ to predict query-document relevance. The system *uogTr* linearly combines a SciBert model^17^ trained on the medical queries of MSMarco^15^ and SciColBERT. The system *unique_ptr* leverages synthetic query generation^39^ for training data augmentation. RRF enables easy merging of ideas. It would be straightforward for CO-Search to be extended to benefit from these ideas: synthetic query generation could augment the SBERT training tuples shown in Fig. 1; the outputs of both a medically fine-tuned SciBert model, or a transformer fine-tuned on the MedMARCO data, could be joined with our own output via RRF.

**Table 4:** Comparison to top automatic runs, using judged documents.

System	Bpref	MAP	P@5	P@10	nDCG@10
covidex	0.5052	0.4739	**0.9040**	**0.8900**	**0.8325**
uogTr	0.4933	0.4580	**0.9040**	0.8720	0.7918
unique_ptr	**0.5364**	**0.5100**	0.8680	0.8380	0.7746
CO-Search	0.5253	0.4884	0.8760	0.8420	0.7567

We compare the performance of CO-Search against the top three systems from Round 5, in the automatic category, using various metrics. The bolded numbers indicate the top-scoring system for the given column’s metric. As can be seen, different systems exhibit different strengths—no single system achieves the highest score across all metrics.

From Round 5, the two topics on which CO-Search performs best, as ranked by Bpref, are “what kinds of complications related to COVID-19 are associated with diabetes” and “are patients taking Angiotensin-converting enzyme inhibitors (ACE) at increased risk for COVID-19?”. Conversely the system performs worst on “what are the guidelines for triaging patients infected with coronavirus?” and “what causes death from Covid-19?”. This is likely due to the hybrid semantic-syntactic nature of the system. The keyword models allow the system to focus in on important words like “diabetes” and “angiotensin”, while the semantic SBERT model would focus on broader meanings inherent in pieces of the text such as “complications..associated with...”. Note that the worst-performing topics lack the obvious keywords of the first.

The semantic search capability of CO-Search allows it to disambiguate between subtle variations in word ordering that, in biological contexts, result in critically different meanings (e.g. “What regulates expression of the ACE2 protein?” vs. “What does the ACE2 protein regulate?”), maximizing its utility to the medical and scientific communities in a time of crisis. Key to the fair evaluation of the system is considering the general use case (all IR systems, all documents), and a specific use case (automatic systems, judged documents).

This work is intended as a tool to support the fight against COVID-19. In this time of crisis, tens of thousands of documents are being published, only some of which are scientific, rigorous, and peer-reviewed. This may lead to the inclusion of misinformation and the potential rapid spread of scientifically disprovable or otherwise false research and data. People on the front lines—medical practitioners, policy makers, etc.—are time-constrained in their ability to parse this corpus, which could impede their ability to approach the returned search results with the appropriate levels of skepticism and inquiry available in less exigent circumstances. Coronavirus-specialized search capabilities are key to making this wealth of knowledge both useful and actionable. The risks are not trivial, as decisions made based on returned, incorrect, or demonstrably false results might jeopardize trust or public health and safety. The authors acknowledge these risks, but believe that the overall benefits to researchers and to the broader COVID-19 research agenda outweigh the risks.

## Methods

### Evaluation metrics

Below we define key metrics in evaluation. Throughout this work we adopt the standard convention that m@N refers to an evaluation using metric *m*, and the top N retrieved documents.

*Precision (P):*

1 $$P@N=\frac{| \{\,{\mathrm{relevant}}\,{\mathrm{documents}}\, {\mathrm{in}}\, {\mathrm{top-N}}\,\}| }{N}$$

*nDCG*: For position *i* ∈ {0, 1, . . . , *N*}, the nDCG of a retrieved set of documents over *Q* queries is given by

2 $$\,{\mathrm{nDCG}}@N=\frac{1}{Q}\mathop{\sum }\limits_{q = 1}^{Q}\frac{{\mathrm{DCG}}_{p}^{(q)}}{{\mathrm{IDCG}}_{p}^{(q)}}\,{\mathrm{, with}}\,\ {\mathrm{DCG}}_{p}^{(q)}={\mathrm{rel}}_{1}^{(q)}+\mathop{\sum }\limits_{i = 2}^{N}\frac{{\mathrm{rel}}_{i}^{(q)}}{{{\mathrm {log}}}_{2}(i)}$$

where ${\,\text{rel}\,}_{i}^{(q)}$ denotes the relevance of entry *i*, ranked according to query *q*. IDCG denotes the ideal and highest possible DCG. In the limit of perfect annotations, nDCG performs reliably in measuring search engine performance. Since it treats non-annotated documents as incorrect (rel_*i*_ evaluates to zero), it is less reliable for datasets with incomplete annotations.

*MAP***:** The average precision (AP) of a retrieved document set is defined as the integral over the normalized precision-recall curve of the set’s query. MAP is defined as the mean AP over all queries:

3 $${\text{MAP}}\,=\frac{1}{Q}\mathop{\sum}\limits_{q = 1}^{Q}\mathop{\int}\nolimits_{0}^{1}{P}_{q}(R){\mathrm{d}}R$$

where *R* is recall, *P*_*q*_ is precision as a function of recall, for a particular query. Note that, as in the case of nDCG, MAP penalizes search engines that yield accurate but unique (i.e. non-annotated) results, since non-annotated documents are treated as irrelevant by *P*.

*Bpref*: Bpref strictly uses information from judged documents. It is a function of how frequently relevant documents are retrieved before non-relevant documents. In situations with incomplete relevance judgments (most IR datasets) it is more stable than other metrics, and it is designed to be robust to missing relevance judgments. It gives roughly the same results with incomplete judgments as MAP would give with complete judgments^38^. It is defined as

4 $${\mathrm{Bpref}}\,=\frac{1}{R}\mathop{\sum }\limits_{r = 1}^{R}1-\frac{| n\ \,{\mathrm{ranked}}\, {\mathrm{higher}}\, {\mathrm{than}}\,r| }{R}$$

where *R* is the number of judged relevant documents, *r* is a relevant retrieved document, *n* is one of the first *R* irrelevant retrieved documents, and non-judged documents are ignored.

### Document indexing

We train the SBERT model of the indexing step with cross-entropy loss, Adam optimization^40^ with a learning rate of 2e–5, a linear learning rate warm-up over 10% of the training data, and a default pooling strategy of MEAN (see Fig. 1a).

### Document retrieval

At runtime, the retrieval step takes an input query, embeds it using SBERT, computes approximate nearest neighbors over the SBERT paragraph embeddings, and returns a set of paragraphs, together with each paragraph’s cosine similarity to the query. TF-IDF and BM25 take as input queries and documents, returning vectors $t\in {{\mathbb{R}}}^{M}$ and $b\in {{\mathbb{R}}}^{M}$ such that *t*_*i*_ = TF-IDF(query, document *i*), *b*_*i*_ = BM25(query, document *i*), and *M* is the size of the document corpus. We build a Lucene index with BM25 retrieval function with default parameters of *k*1 = 1.2, *b* = 0.75 in the Anserini IR toolkit. The formula for TF-IDF is given by

5 $${\mathrm{TF{\hbox{-}}IDF}}\,(t,d)=\,{\mathrm{tf}}\,(t,d)\left({\mathrm{log}}\frac{1+n}{1+\,{\mathrm{df}}\,(t)}+1\right)$$

where tf(*t*, *d*) is the term frequency—the number of times term *t* appears in document *d*—and df(*t*) is the document frequency—the number of documents in the set that contain term *t*. We use the scikit-learn^41^ version of TF-IDF, with a vocabulary size of 13,000, a max document frequency of 0.5, a minimum document frequency of 3, and L2 normalization^42^ of the vectors computed from Eq. (5), above.

The SBERT and TF-IDF scores are combined linearly. For document *d* (containing paragraphs *p*), and query *q*, with subscript *es* denoting an SBERT embedding, their combination *C* is given by

6 $$C(q,d)=\mu \mathop{\max }\limits_{p\in d}[\cos ({p}_{{\mathrm{es}}},{q}_{{\mathrm{es}}})]+(1-\mu )\,{\mathrm{TF{\hbox{-}}IDF}}\,(q,d)$$

This induces a ranking ${R}_{{\mathrm {C}}}^{q}$ on the documents, which is then combined with the BM25-induced ranking ${R}_{{\mathrm {B}}}^{q}$ using reciprocal ranked fusion^31^, to obtain a final retrieved ordering:

7 $${\mathrm {RRF}}(q,d)=\frac{1}{k+{R}_{{\mathrm {C}}}^{q}(d)}+\frac{1}{k+{R}_{{\mathrm {B}}}^{q}(d)}$$

In practice, we find that the constants *μ* = 0.7 and *k* = 60 yield good results. Future work could consider using a learned layer to attend over semantic embeddings and keyword vectors, given the query.

### Document re-ranking

Re-ranking combines the RRF scores of the retrieved documents with the outputs of the QA engine and the summarizer. We define *Q* to measure the degree to which a document answers a query:

8 $$Q(q,d)=1.{1}^{N}\,{\text{, with}}\,N=\mathop{\sum }\limits_{a\in A(q)}{\mathbb{1}}(a\in d)$$

where 1(*x*) is the indicator function: 1(*x*) = {1 if *x* is true, 0 otherwise}. The set *A*(*q*) contains the text span outputs of the QA model. We define *S* to measure the degree to which a document summarizes the set of documents retrieved for a query:

9 $$S(q,d)=\frac{1}{2}+\frac{1}{2}\mathop{\max }\limits_{p\in d}cos({p}_{e},M{(q)}_{e})$$

where *M*(*q*)_e_ is the embedded abstractive summary of *q*, summarized across all retrieved documents. Then the final ranking score *R*(*d*, *q*) of a document, for a particular query, is given by

10 $$R(q,d)=S(q,d)\cdot Q(q,d)\cdot {\mathrm {{RRF}}}(q,d)$$

With higher scores indicating better matches. In essence, rank score *R* is determined by letting *S* and *Q* modulate the retrieval score of a query–document pair.

*Question-Answering*: We follow the HotPotQA setup^32^ and all model parameters contained therein. We use paragraphs with high TF-IDF scores for the given query as negative examples for the sequential paragraph selector. The original beam search is modified to include paragraph diversity and avoid extracting the same answers from different paths.

*Abstractive summarization*: We extend the original GPT-2 model by adding a cross-attention function alongside every existing self-attention function. We constrain the cross-attention function to attend strictly to the final layer outputs of the encoder. We use the base models and hyperparameters of Wolf et al.^43^, with 12 layers, 768-dimensional activations in the hidden layers, and 12 attention heads. The model is pre-trained using self-supervision with a gap-sentence generation objective^44^, where we select a random source sentence per document, replace it with a special mask token in the input 80% of the time, and use that sentence as a prediction target in all cases. We then finetune the model with single-document supervised training, using the first 512 tokens of CORD-19 documents after the abstract as input, and the first 300 tokens of the abstract as target output.

Abstracts are split into five groups based on the number of tokens: <65, 65–124, 125–194, 195–294, >295. During training, a special token is provided to specify the summary length in these five categories. At inference time, the model is initialized to output summaries of token lengths <65 in order to generate more concise summaries.

To adapt the model to operate on multiple retrieved paragraphs from different documents, we concatenate the first four sentences of the retrieved paragraphs until they reach an input length of 512 tokens, then feed this into the summarization model.

### Reporting summary

Further information on research design is available in the Nature Research Reporting Summary linked to this article.

## Supplementary information

Reporting Summary

## Data Availability

All data used in this study was taken from the COVID-19 Open Research Dataset Challenge^9^, and is publicly available. The aggregated data analyzed in this study will be made available upon reasonable request.

## Code availability

The code used in this study will be made available upon reasonable request.
